# Fatal Co-infections with SARS-CoV-2 and *Legionella pneumophila*, England

**DOI:** 10.3201/eid2711.204121

**Published:** 2021-11

**Authors:** Victoria J. Chalker, Hugh Adler, Robert Ball, Falguni Naik, Jessica Day, Baharak Afshar, Amit K. Amin

**Affiliations:** United Kingdom Health Security Agency, London, UK (V.J. Chalker, F. Naik, B. Afshar);; St. Helen’s & Knowsley Teaching Hospitals National Health Service Trust, St. Helen’s, UK (H. Adler, R. Ball);; London Northwest University Healthcare National Health Service Trust, London (A.K. Amin)

**Keywords:** respiratory infections, severe acute respiratory syndrome coronavirus 2, SARS-CoV-2, SARS, COVID-19, coronavirus disease, zoonoses, viruses, coronavirus, bacteria, Legionella pneumophila, England

## Abstract

Both *Legionella pneumophila* and severe acute respiratory syndrome coronavirus 2 (SARS-CoV-2) can cause pneumonia. *L. pneumophila* is acquired from water sources, sometimes in healthcare settings. We report 2 fatal cases of *L. pneumophila* and SARS-CoV-2 co-infection in England. Clinicians should be aware of possible *L. pneumophila* infections among SARS-CoV-2 patients.

Legionnaires’ disease, caused by *Legionella* bacteria, is a factor in community and healthcare acquired pneumonia. *Legionella* infection occurs from manmade water sources, including water aerosolized from cooling towers, spa pools, and water features, and from plumbing in hotels, workplaces, and healthcare facilities (*1*), where patients can be more susceptible to infection (*1*). 

Severe acute respiratory syndrome coronavirus 2 (SARS-CoV-2) causes coronavirus disease (COVID-19), which also can cause pneumonia. Clinically differentiating Legionnaires’ disease from COVID-19 requires laboratory diagnostics, such as urine antigen testing, PCR, and culture. The clinical focus on SARS-CoV-2 potentially causes underdiagnosis of *L. pneumophila* because clinicians might not suspect or investigate the bacterium, but co-infections have been reported. Documented co-infections in COVID-19 patients include human metapneumovirus (*2*), influenza (*3*), *Chlamydia pneumoniae*, *Mycoplasma pneumoniae*, non–COVID-19 coronavirus, enterovirus, rhinovirus, parainfluenza, and respiratory syncytial virus (*4*), and *L. pneumophila* in a case associated with a cruise ship (*5*). Rapid identification of co-infections is essential for managing and treating severe COVID-19 cases (*6*). We describe 2 cases of SARS-CoV-2 and *L. pneumophila* co-infection in patients admitted to hospitals in the United Kingdom.

In February 2020, a female patient >65 years of age was admitted in Addisonian crisis. She was discharged to home but was readmitted to the same ward 8 days later with pneumonia. Her chest radiograph demonstrated minor bibasal opacity. The patient was prescribed amoxicillin/clavulanate and clarithromycin at admission for suspected bacterial infection; only clarithromycin would be effective against *Legionella* infection. No *L. pneumophila* nor SARS-CoV-2 testing was performed at admission and blood culture showed no growth. After initial clinical improvement, the patient’s respiratory status deteriorated on day 10 and her chest radiograph showed extensive bilateral infiltrates. She tested positive for *L. pneumophila* by BinaxNOW Legionella Urinary antigen test (Alere, https://immuview.com) and for SARS-CoV-2 by PCR of nose and throat swab samples. *L. pneumophila* was confirmed by using Legionella Urinary Antigen EIA (Bartels, https://www.trinitybiotech.com) and Rapid Test Kit BinaxNOW Enzyme Immunoassay (EIA; Alere) on urine. No confirmatory lower respiratory samples were obtained. Despite antimicrobial drug and supportive treatment, the patient did not improve and was transitioned to palliative measures. She died 20 days after admission. We used culture to test water from all outlets on the ward; all were negative for *L. pneumophila* serogroup 1. We could not determine whether *L. pneumophila* and SARS-CoV-2 co-infection occurred in the community before hospital admission or in the hospital setting. 

Another co-infection occurred in a woman >80 years of age with a history of hypertension and chronic kidney disease who resided with her family in the community. She was admitted with dyspnea, hypoxia, and acute-on-chronic kidney injury in April 2020. Her chest radiograph demonstrated bilateral mid- and lower-zone consolidation, predominantly peripheral. The patient was prescribed amoxicillin/clavulanate and clarithromycin for suspected bacterial infection. The patient deteriorated over the subsequent 24 hours with progressive hypoxia despite maximal oxygen therapy. A nasal swab collected at admission tested positive for SARS-CoV-2 by PCR. Blood cultures collected at admission were negative. Per guidelines for testing for pneumonia, urine collected on day 2 after admission tested positive by BinaxNOW Legionella Urinary Antigen Test (Alere) and was confirmed with EIA as in the prior case; urinary pneumococcal antigen test was negative. No lower respiratory samples for confirmatory *Legionella* culture and typing were obtained. The treatment strategy was transitioned to palliative measures, and she died 5 days after admission. We assume this patient acquired both *L. pneumophila* and COVID-19 in the community, but she had no apparent epidemiologic risk for *L. pneumophila*, such as travel.

Our study is limited by the low number of cases and the lack of lower respiratory specimens, which prevented confirmation and identification of *L. pneumophila* infection by PCR or culture. Nonetheless, *Legionella* testing of patients and water systems should not be neglected during the SARS-CoV-2 pandemic. Healthcare facilities and clinicians should continue to adhere to recommended protocols for *L. pneumophila* infection prevention and diagnosis. 

Even during the COVID-19 pandemic, patients are at risk for *L. pneumophila* infection in community and healthcare settings. Because periods of water system disuse can permit *Legionella* to grow and increase risk for infection (*1*), pandemic measures, such as temporary closure and reopening of buildings, could increase risk for *Legionella* exposure. Healthcare facilities should follow national guidance for managing *Legionella* during the COVID-19 pandemic and consider publications from the European Society for Clinical Microbiology and Infectious Disease European Study Group for *Legionella* Infections (*7*). 

Hospital-acquired *L. pneumophila* cases and outbreaks can have higher fatality rates than community-acquired single cases (*8*). Recent data indicates bacterial co-infection in SARS-CoV-2 cases is uncommon in patients newly admitted to the hospital (*9*). However, effects of *L. pneumophila* co-infection on COVID-19 mortality rates is not yet known. Large outbreaks might be missed because of reduced testing or less consideration for *L. pneumophila* infection in differential diagnosis. Clinicians should maintain *Legionella* testing and conduct patient investigations where clinically indicated during the pandemic.

In conclusion, patients with SARS-CoV-2 might be at increased risk for other community- or healthcare-acquired infections. Clinicians should be aware of possible *L. pneumophila* infections among SARS-CoV-2 patients.
